# Maternal Exposure to Fine Particulate Matter and Its Chemical Components Increasing the Occurrence of Gestational Diabetes Mellitus in Pregnant Japanese Women

**DOI:** 10.31662/jmaj.2022-0141

**Published:** 2022-09-26

**Authors:** Takehiro Michikawa, Seiichi Morokuma, Shin Yamazaki, Ayako Yoshino, Seiji Sugata, Akinori Takami, Kazushige Nakahara, Shinji Saito, Junya Hoshi, Kiyoko Kato, Hiroshi Nitta, Yuji Nishiwaki

**Affiliations:** 1 Department of Environmental and Occupational Health, School of Medicine, Toho University, Tokyo, Japan; 2 Department of Health Sciences, Graduate School of Medical Sciences, Kyushu University, Fukuoka, Japan; 3 Health and Environmental Risk Division, National Institute for Environmental Studies, Ibaraki, Japan; 4 Regional Environment Conservation Division, National Institute for Environmental Studies, Ibaraki, Japan; 5 Department of Obstetrics and Gynaecology, Graduate School of Medical Sciences, Kyushu University, Fukuoka, Japan; 6 Tokyo Metropolitan Research Institute for Environmental Protection, Tokyo, Japan

**Keywords:** chemical element, fine particle, gestational diabetes mellitus, organic carbon, pregnancy

## Abstract

**Introduction:**

PM_2.5_ exposure is a suspected risk factor for diabetes. It is hypothesized that maternal PM_2.5_ exposure contributes to the development of gestational diabetes mellitus (GDM). The association between PM_2.5_ exposure and GDM is controversial and limited evidence is available for the exposure to PM_2.5_ chemical components. We investigated the association between maternal exposure to total PM_2.5_ mass and its components, particularly over the first trimester (early placentation period), and GDM.

**Methods:**

Data were obtained from the Japan Perinatal Registry Network database, which includes all live births and stillbirths after 22 weeks of gestation at 39 cooperating hospitals in 23 Tokyo wards (2013-2015). At one fixed monitoring site, we performed daily filter sampling of fine particles and measured daily concentrations of carbon and ion components. The average concentrations of total PM_2.5_ and its components over the 3 months before pregnancy and the first (0-13 gestational weeks) and second (14-27 gestational weeks) trimesters were calculated and assigned to each woman. We estimated the odds ratios (ORs) of GDM in a multilevel logistic regression model.

**Results:**

Among 82,773 women (mean age at delivery = 33.7 years) who delivered singleton births, 3,953 (4.8%) had GDM. In the multiexposure period model, an association between total PM_2.5_ exposure and GDM was observed for exposure over the first trimester (OR per interquartile range (IQR = 3.63 μg/m^3^) increase = 1.09; 95% confidence interval (CI) = 1.02-1.16), but not for the 3 months before pregnancy or the second trimester. For PM_2.5_ components, only organic carbon exposure over the first trimester was positively associated with GDM (OR per IQR (0.51 μg/m^3^) increase = 1.10; 1.00-1.21).

**Conclusions:**

This is the first evidence that exposure to total PM_2.5_ and one of its components, organic carbon, over the first trimester increases GDM occurrence in Japan.

## Introduction

Although the underlying mechanisms have not been fully elucidated, a causal association between exposure to fine particulate matter (PM_2.5_, which passes through a size-selective inlet with a 50% cut-off level of 2.5 μm in aerodynamic diameter) and cardiorespiratory disease seems to be internationally accepted ^(1)^. PM_2.5_ exposure is also associated with glycemia and insulin resistance and is a suspected risk factor for diabetes ^(2)^. It is hypothesized that maternal exposure to PM_2.5_ contributes to the development of gestational diabetes mellitus (GDM). However, the association between PM_2.5_ exposure and GDM is controversial ^(3), (4)^, and there is limited evidence about whether exposure to specific chemical components of PM_2.5_ leads to GDM ^(5), (6), (7), (8), (9), (10)^.

There are inconsistent findings regarding the periods during which PM_2.5_ exposure is related to GDM etiology. One systematic review and meta-analysis including relevant studies published until August 2019 reported that PM_2.5_ exposure in the second trimester increased the occurrence of GDM ^(4)^. Another meta-analysis including studies published until September 2019 reported that there was no specific exposure period and that the point estimates of the association between exposure in each period (prepregnancy, the first trimester, and second trimester) and GDM showed nonsignificant elevated odds ^(3)^. GDM is considered to precede the occurrence of preeclampsia related to abnormal placentation in early pregnancy ^(11), (12)^. In an experiment using a human trophoblast cell line from the first trimester, hyperglycemia triggered trophoblast secretion of inflammatory cytokines, suggesting that excess glucose leads to trophoblast dysfunction and inhibits adequate placentation ^(13)^. Therefore, if exposure to PM_2.5_ was causally associated with GDM, we hypothesized that exposure during early pregnancy (i.e., the first trimester) would be important.

Based on this background, we investigated the association between maternal exposure to total PM_2.5_ and its components, particularly over the first trimester, and GDM among pregnant Japanese women who resided in Tokyo, an international megacity.

## Materials and Methods

### Study population

This study was conducted in 23 Tokyo wards that generally fell within a circle with a radius of 20 km, with approximately 9.3 million people (as of 2015) ^(14)^ living in a land area of roughly 627 km^2^. The average background PM_2.5_ concentration over the study period (2013-2015) was 15.9 μg/m^3^, which was slightly higher than the annual air quality standard in Japan (15 μg/m^3^) ^(15), (16)^.

The Japan Society of Obstetrics and Gynecology manages the Japan Perinatal Registry Network database, a voluntary ongoing registry of obstetric facilities (mainly university hospitals and local general hospitals) ^(17)^. Each collaborating facility submits data annually, which include anonymized information on maternal age, height, weight, parity, gestational age, smoking and alcohol consumption patterns, infertility treatment, medical history, diagnosis of obstetric complications, mode of delivery, and neonatal records, on all the live births and stillbirths after 22 weeks of gestation via a standardized electronic form. The Perinatal Committee of the Japan Society of Obstetrics and Gynecology checks the quality of the submitted data and requests data correction when needed.

From the Japan Perinatal Registry Network database, we obtained the data on 89,417 registered births (including multiple births) from 39 cooperating facilities in 23 Tokyo wards between January 2013 and December 2015. These registered births accounted for roughly 40% of the total births in the 23 studied wards. Initially, we selected 85,476 singleton births (85,476 women) without missing information on maternal age at delivery. Then, we excluded 366 women with overt diabetes during pregnancy including some cases of pregestational diabetes, 1,658 women who delivered at hospitals near their parents’ home, rather than near their own home (a Japanese custom called *satogaeri*) to avoid exposure misclassification, and 679 women who delivered at 22-27 weeks of pregnancy to define exposure over the second trimester. Finally, 82,773 women were included in this study. The PM_2.5_ chemical component measurements only began on 1 April 2013 (3 months after the beginning of the study); therefore, component exposure was only assigned to 67,136 women whose first trimester fell within this later period.

The study protocol was approved by the Ethics Committee of Faculty of Medicine, Toho University [A20024_A18049].

### PM_2.5_ exposure

The PM_2.5_ measurement method and locations of the monitoring sites and hospitals are described in our previous publications ^(18), (19)^. Briefly, PM_2.5_ components were measured at the Tokyo Metropolitan Research Institute for Environmental Protection (TMRIEP) (35.7°N, 139.8°E). The measurements from there likely reflect the typical urban background concentrations of air pollutants in the 23 studied Tokyo wards ^(20)^. On a rooftop of the TMRIEP, we used an FRM-2000 sampler (Rupprecht & Patashnick, Albany, NY, USA) and a quartz-fiber filter (47 mm diameter, 2500 QAT-UP; Pall Life Sciences, Port Washington, NY, USA) to collect fine particles daily (from 10:00 a.m. to 9:00 a.m. of the next day) between April 2013 and the end of the study period. This followed the Federal Reference Methods of the US Environmental Protection Agency ^(21)^. According to the standardized protocol of the Ministry of Environment, Japan ^(22)^, the daily concentrations of total carbon, including organic carbon (OC) and elemental carbon (EC), were determined by thermal-optical reflectance methods with a thermal-optical carbon analyzer (OCEC Carbon Aerosol Analyzer; Sunset Laboratory Inc., Tigard, OR, USA). In addition, ions, including nitrate, sulfate, ammonium, chloride, sodium, potassium, and calcium, were analyzed using an ion chromatograph (Dionex ICS-5000; Thermo Fisher Scientific Inc., Waltham, MA, USA), and their daily concentrations were estimated.

Hourly measurements of total PM_2.5_ mass (β-ray absorption method) and ozone concentrations (ultraviolet absorption method) at an urban background monitoring station (Harumi monitoring station, 35.4°N, 139.5°E, roughly 5 km west of the TMRIEP) were obtained from the Japan National Institute for Environmental Studies’ atmospheric environment database. The daily mean concentrations of total PM_2.5_ were estimated when there were daily measurements more than or equal to 20 h, and the daily mean concentrations of ozone for a maximum of 8 h were also estimated. As we previously reported ^(18)^, the daily mean concentrations of total PM_2.5_ and maximum 8 h mean concentrations of ozone at the Harumi monitoring station well correlated with those at eight other urban background monitoring stations within the 23 Tokyo wards. We, then, estimated the coefficient of divergence to check the similarities between the measurements at the Harumi station and those at the other stations within the 23 wards. The coefficient of divergence was defined as (*xih*−*xik*)/(*xih+xik*) ^(23)^, where *xih* and *xik* represent the average concentrations of total PM_2.5_ or ozone (i) at the Harumi station (h) and other stations (k). If the concentration at the Harumi station was in absolute agreement with that at another station, the coefficient of divergence was zero. Over the study period, the average of eight coefficients of divergences was 0.04 for total PM_2.5_ and 0.05 for ozone, suggesting little divergence. Therefore, we assumed that the concentrations of total PM_2.5_ and ozone were spatially homogenous within the 23 Tokyo wards. Additionally, we collected the daily mean ambient temperature from the database of the Japan Meteorological Agency.

Based on the assumption that pollutant concentrations were homogenous within the 23 Tokyo wards, we assigned the daily pollutant concentrations measured at the Harumi station and TMRIEP to all the women. Depending on the period of the first trimester (0-13 weeks of gestation), which was estimated from the birth date and gestational age based on ultrasound findings during early pregnancy, the average concentration of each pollutant over the first trimester was calculated. We also calculated the average concentrations over the 3 months before pregnancy and the second trimester (14-27 weeks of gestation), and used these as exposure variables, similar to previous studies ^(6), (24)^. Incidentally, the obtained data did not include the residential addresses (e.g., postal codes) of the women.

### GDM

Data obtained from the Perinatal Registry Network database included information on GDM diagnosed by attending physicians. In Japan, a 75 g oral glucose tolerance test was performed in the second or third trimester, and GDM was defined if the plasma glucose level at three time points was higher than the following cut-off values: fasting ≥92 mg/dL (5.1 mmol/L), 1 h ≥180 mg/dL (10.0 mmol/L), and 2 h ≥153 mg/dL (8.5 mmol/L) ^(25)^. Although the revised diagnostic criteria of hyperglycemic disorders in pregnancy were presented in August 2015 (the final year of the study period), the criteria for GDM were not changed ^(26)^. GDM was treated as a dichotomous variable.

### Statistical methods

We examined the association between exposure to total PM_2.5_ and its components and GDM using a multilevel logistic regression model with the hospital as a random effect. The majority of women were assumed to reside near their delivery hospitals ^(27)^; therefore, participants who delivered at the same hospital were considered to potentially have similar social environmental characteristics, and the hospital was chosen as a neighborhood-level social environmental factor. An assumption of a linear association between PM_2.5_ exposure and GDM was not inferior to another assumption of a nonlinear association. The odds ratios (ORs) and 95% confidence intervals (CIs) per interquartile range (IQR) increase in the average pollutant concentrations were estimated. We explored the association between total PM_2.5_ exposure over three periods (the 3 months before pregnancy, and the first and second trimesters) and GDM separately (single-exposure period model), after adjustment for maternal age at delivery (<25, 25-29, 30-34, ≥35 years), the season of conception (spring, summer, autumn, winter), and potential confounding factors related to diabetes and/or GDM ^(28), (29)^, as follows: parity (0, ≥1, missing), smoking habits (yes, no, missing), alcohol drinking (yes, no, missing), prepregnancy body mass index (BMI) (<18.5, 18.5-24.9, ≥25 kg/m^2^, missing), past history of GDM (yes, no), and infertility treatment (no, ovarian stimulation/artificial insemination of sperm from husband, assisted reproductive technology). Infertility treatment is treated as a variable related to polycystic ovary syndrome, which is a suspected risk factor for GDM ^(30)^. To evaluate which exposure period showed a clear association with GDM, we simultaneously included three exposure periods in the model (multiexposure period model). Moreover, we performed several sensitivity analyses to check whether the observed association was robust. First, we restricted the analysis to nonsmokers during pregnancy, nonalcohol drinkers during pregnancy, women with prepregnancy BMI <25 kg/m^2^, and women with spontaneous pregnancy, to minimize residual confounding. Second, we adjusted for ozone concentrations and ambient temperature ^(31)^ (a 5-knot natural cubic spline) over the same period as the PM_2.5_ exposure. Finally, we restricted the analysis to women who delivered in hospitals within 10 km from the monitoring sites to minimize exposure misclassification.

This study focused on exposure over the first trimester. Therefore, we explored whether exposure to PM_2.5_ components over the first trimester was associated with GDM in the single-component model (target component only). After constructing a multiexposure period model, we further adjusted for total PM_2.5_ concentration over the first trimester. For sensitivity analysis, we created a multicomponent model that included other components, one by one. In addition, we examined the association between ozone exposure and GDM in the single-exposure and multiexposure period models, and in the total PM_2.5_- and ambient temperature-adjusted model. All analyses were conducted using the Stata version 16 for Windows (Stata Corporation, College Station, TX, USA).

## Results

The characteristics of the 82,773 women (mean age at delivery = 33.7 years), of whom 3,953 (4.8%) were diagnosed with GDM, are shown in Table 1. The summary statistics of pollutant exposure are presented in Table 2. The median concentration of total PM_2.5_ over the first trimester was 16.1 μg/m^3^ (IQR = 3.63 μg/m^3^). Five components, namely, OC, EC, nitrate, sulfate, and ammonium, contributed to approximately 60% of the total PM_2.5_. Table S1 provides the Pearson’s correlation coefficients of the concentrations of PM_2.5_ and its components over the different exposure periods (the 3 months before pregnancy, and the first and second trimesters).

**Table 1.** Characteristics of 82,773 Women Who Delivered at the Cooperating Hospitals in the 23 Tokyo Wards in 2013-2015.

Variables		n*	%
Maternal age at delivery (years)	<25	3,205	3.9
	25-29	13,420	16.2
	30-34	28,359	34.3
	≥35	37,789	45.6
Parity	0	49,978	60.4
	≥1	32,778	39.6
Smoking during pregnancy	No	65,218	96.4
	Yes	2,416	3.6
Alcohol drinking during pregnancy	No	57,553	96.0
	Yes	2,395	4.0
Prepregnancy body mass index (kg/m^2^)	<18.5	13,772	19.6
	18.5-24.9	51,449	73.2
	≥25.0	5,066	7.2
Infertility treatment	No	69,624	84.1
	Ovarian stimulation/artificial insemination by sperm from husband	5,276	6.4
	Assisted reproductive technology	7,873	9.5
Past history of gestational diabetes mellitus	No	82,712	99.9
	Yes	61	0.1
Season of conception	Spring (March-May)	19,969	24.1
	Summer (June-August)	20,285	24.5
	Autumn (September-November)	21,443	25.9
	Winter (December-February)	21,076	25.5

*Numbers in subgroups do not equal the overall number because of missing data.

**Table 2.** Summary Statistics of Pollutant Exposures in the 23 Tokyo Wards.

	Exposure over the first trimester (0-13 weeks of gestation)					Exposure over the 3 months before pregnancy	Exposure over the second trimester (14-27 weeks of gestation)
	No. of women	Mean (SD)	Percentile			Mean (SD)	Mean (SD)
			25	50	75		
Total PM_2.5_ (μg/m^3^)	82,773	16.8 (2.6)	14.9	16.1	18.5	16.7 (2.8)	16.8 (2.6)
PM_2.5_ components (μg/m^3^)*
Total carbon	67,136	4.0 (0.5)	3.7	4.1	4.3	3.9 (0.6)	3.9 (0.6)
OC	67,136	2.7 (0.4)	2.4	2.7	2.9	2.6 (0.5)	2.6 (0.4)
EC	67,136	1.3 (0.2)	1.2	1.3	1.4	1.3 (0.2)	1.3 (0.2)
Nitrate	67,136	1.4 (0.8)	0.6	1.3	2.1	1.2 (0.8)	1.3 (0.8)
Sulfate	67,136	2.8 (1.0)	1.9	2.8	3.6	2.8 (1.1)	2.9 (1.0)
Ammonium	67,136	1.5 (0.3)	1.2	1.5	1.8	1.4 (0.3)	1.5 (0.3)
Chloride	67,136	0.20 (0.13)	0.08	0.15	0.34	0.19 (0.15)	0.19 (0.13)
Sodium	67,136	0.15 (0.03)	0.12	0.15	0.17	0.15 (0.03)	0.15 (0.03)
Potassium	67,136	0.07 (0.01)	0.06	0.07	0.08	0.07 (0.02)	0.07 (0.01)
Calcium	67,136	0.07 (0.02)	0.06	0.07	0.09	0.07 (0.02)	0.07 (0.02)
Ozone (ppb)	82,773	35.9 (7.4)	29.3	35.0	42.7	35.7 (7.5)	36.7 (7.6)
Ambient temperature (°C)	82,773	16.5 (7.0)	9.7	16.7	23.3	16.9 (7.2)	16.7 (6.9)

EC, elemental carbon; IQR, interquartile range; OC, organic carbon; SD, standard deviation. *The PM_2.5_ chemical component measurements only began on 1 April 2013 (3 months after the beginning of the study); therefore, we only assigned these measurements to the 67,136 women whose first trimester fell within this later period.

The association between exposure to total PM_2.5_ and GDM is shown in Table 3. In the single-exposure period model, exposure over both the first and second trimesters was associated with GDM. However, when we applied the multiexposure period model (which simultaneously included exposure over the 3 months before pregnancy and the first and second trimesters), an association was observed only between exposure over the first trimester and GDM. The ORs per IQR increase in the PM_2.5_ concentration were 1.09 (95% CI = 1.02-1.16) for the first trimester, 0.98 (0.93-1.04) for the 3 months before pregnancy, and 1.04 (0.98-1.11) for the second trimester. For the association between exposure over the first trimester and GDM, sensitivity analyses revealed that the point estimates of ORs were comparable with the main result in the multiexposure period model.

**Table 3.** Odds Ratios (ORs) and 95% Confidence Intervals (CIs) for the Association between Exposure to Total PM_2.5_ Mass and Gestational Diabetes Mellitus.

	No. of women	No. of outcomes	OR per IQR increase (95% CI)*
Exposure over the first trimester (0-13 weeks of gestation)			
Single-exposure period	82,773	3,953	1.09 (1.03-1.16)
Multiexposure period**	82,773	3,953	1.09 (1.02-1.16)
Sensitivity analyses			
Restricted to nonsmokers during pregnancy	65,218	3,185	1.10 (1.02-1.18)
Restricted to nonalcohol drinkers during pregnancy	57,553	3,145	1.11 (1.04-1.19)
Restricted to women with prepregnancy body mass index <25.0 kg/m^2^	65,221	2,710	1.09 (1.01-1.17)
Restricted to women with spontaneous pregnancy	69,624	3,149	1.08 (1.01-1.16)
Additionally adjusted for ozone concentration and ambient temperature over the first trimester	82,773	3,953	1.11 (1.02-1.22)
Restricted to women who delivered in hospitals within 10 km from the monitoring sites	61,436	2,969	1.08 (1.01-1.14)
Exposure over the 3 months before pregnancy			
Single-exposure period	82,773	3,953	1.00 (0.94-1.05)
Multiexposure period**	82,773	3,953	0.98 (0.93-1.04)
Exposure over the second trimester (14-27 weeks of gestation)			
Single-exposure period	82,773	3,953	1.06 (1.00-1.12)
Multiexposure period**	82,773	3,953	1.04 (0.98-1.11)

IQR, interquartile range = 3.63 μg/m^3^ *Adjusted for maternal age, season of conception, parity, smoking, alcohol drinking, prepregnancy body mass index, infertility treatment, and past history of gestational diabetes. **We simultaneously included three exposures in the model.

**Figure 1.**
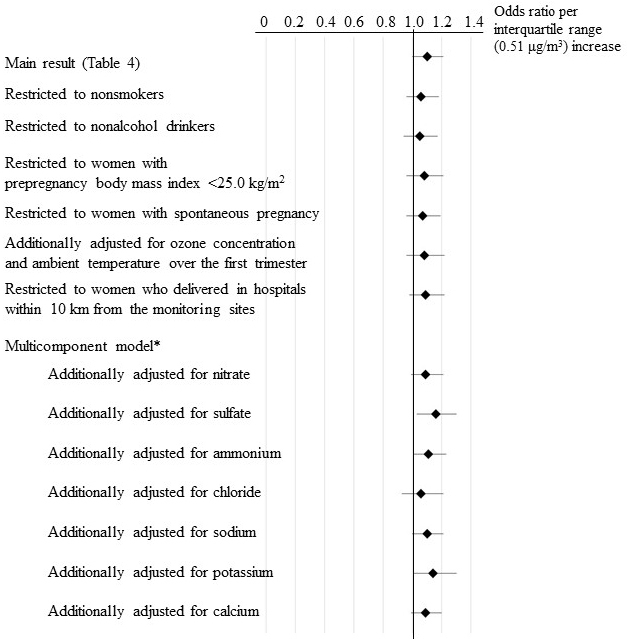
Sensitivity Analysis of the Association between Exposure to Organic Carbon (OC) over the First Trimester (0–13 Weeks of Gestation) and Gestational Diabetes Mellitus. The odds ratios were adjusted for exposure to OC over the 3 months before pregnancy and the second trimester, maternal age, season of conception, parity, smoking, alcohol drinking, prepregnancy body mass index, infertility treatment, past history of gestational diabetes, and total PM_2.5_ concentration over the first trimester. The error bars indicate 95% confidence intervals. *We did not construct a multicomponent model that included the same carbon component (elemental carbon).

The association between exposure to a single PM_2.5_ component over the first trimester and GDM is shown in Table 4. In the multiexposure period model, the OR per IQR increase in the OC concentration (0.51 μg/m^3^) was 1.10 (95% CI = 1.00-1.21). Even after adjustment for exposure to total PM_2.5_ over the first trimester, this positive association persisted (OR = 1.10, 1.00-1.21). Although there was a dispersion of the point estimates of the association between OC exposure and GDM in the sensitivity analyses, the tendency of OC exposure to increase the odds of GDM did not change (Figure 1).

**Table 4.** Association between Exposure to PM_2.5_ Components over the First Trimester (0-13 Weeks of Gestation) and Gestational Diabetes Mellitus in the Single-component Model.

	Model	OR per IQR increase (95% CI)*
Total carbon	Single-exposure period	1.09 (1.04-1.14)
	Multiexposure period**	1.07 (0.98-1.16)
	Additionally adjusted for total PM_2.5_	1.06 (0.97-1.16)
OC	Single-exposure period	1.10 (1.04-1.16)
	Multiexposure period**	1.10 (1.00-1.21)
	Additionally adjusted for total PM_2.5_	1.10 (1.00-1.21)
EC	Single-exposure period	1.06 (1.00-1.12)
	Multiexposure period**	1.04 (0.96-1.12)
	Additionally adjusted for total PM_2.5_	1.03 (0.94-1.12)
Nitrate	Single-exposure period	1.16 (1.05-1.28)
	Multiexposure period**	1.06 (0.92-1.23)
	Additionally adjusted for total PM_2.5_	1.03 (0.88-1.21)
Sulfate	Single-exposure period	1.01 (0.91-1.12)
	Multiexposure period**	0.97 (0.87-1.09)
	Additionally adjusted for total PM_2.5_	0.93 (0.78-1.10)
Ammonium	Single-exposure period	1.15 (1.04-1.28)
	Multiexposure period**	1.02 (0.87-1.19)
	Additionally adjusted for total PM_2.5_	1.10 (0.88-1.38)
Chloride	Single-exposure period	1.20 (1.07-1.34)
	Multiexposure period**	1.12 (0.98-1.28)
	Additionally adjusted for total PM_2.5_	1.19 (0.99-1.43)
Sodium	Single-exposure period	0.99 (0.89-1.10)
	Multiexposure period**	0.97 (0.84-1.13)
	Additionally adjusted for total PM_2.5_	0.98 (0.83-1.15)
Potassium	Single-exposure period	1.05 (0.99-1.10)
	Multiexposure period**	1.03 (0.96-1.09)
	Additionally adjusted for total PM_2.5_	1.02 (0.96-1.09)
Calcium	Single-exposure period	1.03 (0.96-1.10)
	Multiexposure period**	1.01 (0.93-1.10)
	Additionally adjusted for total PM_2.5_	0.99 (0.90-1.09)

CI, confidence interval; EC, elemental carbon; IQR, interquartile range; OC, organic carbon; OR, odds ratio. *Adjusted for maternal age, season of conception, parity, smoking, alcohol intake, prepregnancy body mass index, infertility treatment, and past history of gestational diabetes. IQRs were 0.62 μg/m^3^ for total carbon, 0.51 μg/m^3^ for OC, 0.22 μg/m^3^ for EC, 1.48 μg/m^3^ for nitrate, 1.77 μg/m^3^ for sulfate, 0.57 μg/m^3^ for ammonium, 0.26 μg/m^3^ for chloride, 0.06 μg/m^3^ for sodium, 0.02 μg/m^3^ for potassium, and 0.03 μg/m^3^ for calcium. **We simultaneously included three exposures in the model.

Exposure to ozone over the first and second trimesters was associated with GDM (Table S2). However, when we adjusted for the total PM_2.5_ concentration and ambient temperature over the same period, the lower values of the 95% CI were below unity (OR per IQR (13.40 ppb) increase = 1.11, 95% CI = 0.85-1.45 for the first trimester; OR = 1.17, 0.91-1.51 for the second trimester).

## Discussion

Among pregnant Japanese women, exposure to total PM_2.5_ over the first trimester was positively associated with GDM. Our analyses of PM_2.5_ components showed that OC exposure was consistently associated with elevated ORs for GDM.

We found that an increase in the total PM_2.5_ concentration over the first, but not the second, trimester was associated with an increased occurrence of GDM. Some studies had observed the association with first trimester exposure. A register-based study conducted in Florida between 2004 and 2005 reported that exposure to total PM_2.5_ during the first trimester (mean: 9.7 μg/m^3^) was positively associated with GDM ^(32)^. A birth cohort conducted in Wuhan between 2013 and 2015 showed that the OR of GDM per 10 μg/m^3^ increase in total PM_2.5_ exposure during the first trimester (mean: 103.1 μg/m^3^) was 1.07 (95% CI = 1.02-1.14) ^(33)^. It is understandable that exposure to PM_2.5_ during early pregnancy increases the occurrence of GDM. GDM likely adversely affects placental development, which starts in the first trimester ^(34), (35)^. Abnormal placentation seems to be involved in the occurrence of preeclampsia and GDM is a suspected risk factor for preeclampsia ^(11), (36)^. Placental vascular indices assessed by ultrasound imaging during the first trimester were lower in women with GDM than in those without GDM ^(37)^. Our previous findings suggested that PM_2.5_ exposure affected maternal and fetal health from early pregnancy ^(38)^. However, our findings in the multiexposure period model were inconsistent with those of some previous epidemiological studies in terms of the susceptible period for PM_2.5_ exposure associated with GDM. One study reported that exposure in all the periods examined (preconception, and the first and second trimesters) was associated with GDM ^(6)^. Another study reported an association between preconception PM_2.5_ exposure and GDM ^(39)^. Other studies reported that exposure during the second trimester was associated with a higher occurrence of GDM ^(8), (40)^. The pathway linking PM_2.5_ exposure with GDM may not necessarily involve the placenta.

We found that the adverse effects of total PM_2.5_ exposure in terms of GDM development were partly explained by OC. This finding was consistent with the results of a study conducted in the Pearl River Delta region of China, which collected the birth records of 1,148 pregnant women between 2015 and 2016, and reported that exposure to OC over the first trimester, as analyzed by filter samples, elevated GDM occurrence ^(10)^. OC is one of the main components of PM_2.5_ and is derived from combustion, particularly from residential sources ^(41)^. Although the biological mechanisms underlying the association between OC exposure and GDM are unclear, oxidative stress and inflammation induced by OC exposure may contribute to GDM development ^(42), (43), (44)^. Epidemiological studies reported that a systemic inflammatory marker, C-reactive protein, was associated with an increase in glucose intolerance and insulin resistance ^(45), (46)^. In experimental studies using mice, oxidative stress and/or inflammation appeared to increase vascular insulin resistance and/or impair hepatic glucose metabolism ^(47), (48)^. EC (or black carbon), another carbon component, likely leads to the inflammatory response ^(42), (43), (44)^. However, EC was not associated with GDM in the present study. Some studies reported a positive association between EC exposure over the first trimester and GDM ^(7), (10)^, but other studies did not ^(5), (8), (49)^.

In this study, ozone exposure over any period was not associated with GDM after adjustment for the PM_2.5_ concentration and temperature. Some studies reported that the ORs of GDM decreased with an increase in ozone concentration before and during pregnancy ^(5), (50), (51)^. One study conducted in the United States and two studies conducted in China reported a positive association between ozone exposure during the first and second trimesters and GDM ^(32), (52), (53)^. However, they did not construct a multi-trimester model including both PM_2.5_ concentration and temperature which were associated with adverse pregnancy outcomes ^(54)^. Ozone concentrations differ among studies and the association between ozone exposure and GDM merits further investigation.

The limitations of this study should be acknowledged. Firstly, our exposure assessment relied on measurements from a fixed monitoring site with the assumption that air pollutant concentrations were spatially homogenous within the 23 Tokyo wards studied. Although this assumption seems reasonable for total PM_2.5_ and ozone, it is not always true for all PM_2.5_ components. However, we performed a sensitivity analysis based on the distance of the delivery hospital from the monitoring sites and confirmed the reliability of the positive association between OC exposure and GDM. Secondly, exposure misclassification due to lack of personal exposure measurements was possible. Nevertheless, such misclassification was unlikely to be associated with GDM and, therefore, would tend to result in the underestimation of the association between pollutants exposure and GDM. Thirdly, there was no information on pregestational diabetes in the Perinatal Registry Network database. Although we excluded women with overt diabetes in pregnancy including some cases of pregestational diabetes, the non-GDM population in this study might include women with pregestational diabetes. Finally, because the university and local general hospitals cooperated in registering their birth records in the database, the frequency of GDM tended to be higher in this population (4.8%) than in the general population (2.1% in the Japanese nationwide birth cohort study, which recruited more than 100,000 general pregnant women between 2011 and 2014 ^(55)^). Our target population likely consisted of a vulnerable population that was sensitive to exposure to PM_2.5_. We might have overestimated the odds of development of GDM in relation to PM_2.5_ exposure, although this does not repudiate the association between PM_2.5_ exposure and GDM. Despite these limitations, this study reports the association between maternal exposure to PM_2.5_ and GDM in Japan for the first time. Additionally, we used continuous filter-based measurements of PM_2.5_ components in a megacity, Tokyo, which allowed us to analyze a relatively large sample size.

In conclusion, we found an association between exposure to total PM_2.5_ and one of its components, OC, and GDM. Our findings suggest that PM_2.5_ exposure over the first trimester is linked with GDM.

## Article Information

This article is based on the study, which received the Medical Research Encouragement Prize of The Japan Medical Association in 2021.

### Conflicts of Interest

None

### Sources of Funding

This work was supported by the Medical Research Encouragement Prize of The Japan Medical Association in 2021, and by the Japan Society for the Promotion of Science KAKENHI grant number 18H03388 and 21H03615. The funding sources had no role in the collection, analysis, or interpretation of the data or in the decision to submit the article for publication.

### Acknowledgement

The authors thank Ms. Masami Matsushita and Ms. Emi Yamazaki (Department of Environmental and Occupational Health, School of Medicine, Toho University) for their research assistance. The PM_2.5_ components data were provided by Type II joint research between the National Institute for Environmental Studies and the local environmental research institutes in Japan.

### Author Contributions

T.M. had full access to all the data in the study and takes responsibility for the integrity of the data and the accuracy of the data analysis. T.M. and S.M. participated in the study conception and design. S.M., K.N., and K.K. participated in the outcome data collection/management. A.Y., S. Sugata, and A.T. participated in the exposure data collection/management. S. Saito and J.H. collected the fine particles and analyzed PM_2.5_ chemical components. T.M. analyzed the data. S.Y., H.N., and Y.N. supervised the analysis. All authors interpreted the results. T.M. drafted the manuscript. All the authors participated in the critical revision of the manuscript and approved the final version of the manuscript.

### Approval by Institutional Review Board (IRB)

The study protocol was approved by the Ethics Committee of Faculty of Medicine, Toho University [A20024_A18049].

### Data Availability

Data used in this study were obtained from the Japan Perinatal Registry Network database, which is managed by the Japan Society of Obstetrics and Gynecology. This society allows members to use the data for scientific research but does not allow anyone to share original data publicly.

## Supplement

Supplementary File
