# Supplementary material for: Maternal Exposure to Fine Particulate Matter and Its Chemical Components Increasing the Occurrence of Gestational Diabetes Mellitus in Pregnant Japanese Women
Source: JMA J. 2022 Sep 26;5(4):480–90. doi: 10.31662/jmaj.2022-0141 (PMC9646294; doi:10.31662/jmaj.2022-0141)
Supplement: Supplementary file 1 — Supplementary File [file 2433-3298-5-4-0480-s001.pdf]

Table S1. Pearson's Correlation Coefficients of Concentrations of $PM_{2.5}$ and its Components in the Different Exposure Periods

		First trimester	Three months before pregnancy	Second trimester
Total $PM_{2.5}$	First trimester	1		
	Before pregnancy	0.08	1	
	Second trimester	0.01	-0.36	1
$PM_{2.5}$ components				
Total carbon	First trimester	1		
	Before pregnancy	0.29	1	
	Second trimester	-0.11	-0.02	1
OC	First trimester	1		
	Before pregnancy	0.34	1	
	Second trimester	-0.10	0.00	1
EC	First trimester	1		
	Before pregnancy	0.16	1	
	Second trimester	0.17	-0.03	1
Nitrate	First trimester	1		
	Before pregnancy	0.24	1	
	Second trimester	-0.08	-0.86	1
Sulfate	First trimester	1		
	Before pregnancy	-0.08	1	
	Second trimester	-0.18	-0.71	1
Ammonium	First trimester	1		
	Before pregnancy	-0.24	1	
	Second trimester	-0.29	-0.56	1
Chloride	First trimester	1		
	Before pregnancy	0.14	1	
	Second trimester	-0.11	-0.70	1
Sodium	First trimester	1		
	Before pregnancy	-0.24	1	
	Second trimester	-0.28	-0.62	1
Potassium	First trimester	1		
	Before pregnancy	-0.12	1	
	Second trimester	-0.35	0.42	1
Calcium	First trimester	1		
	Before pregnancy	-0.22	1	
	Second trimester	-0.36	-0.35	1

EC, elemental carbon; OC, organic carbon.

Table S2. Odds Ratios (ORs) and 95% Confidence Intervals (CIs) for the Association between Exposure to Ozone and Gestational Diabetes Mellitus.

	No. of women	No. of outcomes	OR per IQR increase (95% CI)*
Exposure over the first trimester (0–13 weeks of gestation)			
Single-exposure period	82,773	3,953	1.14 (1.02–1.27)
Multiexposure period**	82,773	3,953	1.23 (1.09–1.39)
Further adjusted for total $PM_{2.5}$ concentration and ambient temperature over the first trimester	82,773	3,953	1.11 (0.85–1.45)
Exposure over the 3 months before pregnancy			
Single-exposure period	82,773	3,953	0.92 (0.83–1.02)
Multiexposure period**	82,773	3,953	1.06 (0.93–1.21)
Further adjusted for total $PM_{2.5}$ concentration and ambient temperature over the 3 months before pregnancy	82,773	3,953	1.03 (0.85–1.25)
Exposure over the second trimester (14–27 weeks of gestation)			
Single-exposure period	82,773	3,953	1.17 (1.04–1.31)
Multiexposure period**	82,773	3,953	1.27 (1.11–1.47)
Further adjusted for total $PM_{2.5}$ concentration and ambient temperature over the second trimester	82,773	3,953	1.17 (0.91–1.51)

IQR, interquartile range = 13.40 ppb

*Adjusted for maternal age, season of conception, parity, smoking, alcohol drinking, prepregnancy body mass index, infertility treatment, and past history of gestational diabetes.

**We simultaneously included three exposures in the model.
